# Individuals Underestimate Moderate and Vigorous Intensity Physical Activity

**DOI:** 10.1371/journal.pone.0097927

**Published:** 2014-05-16

**Authors:** Karissa L. Canning, Ruth E. Brown, Veronica K. Jamnik, Art Salmon, Chris I. Ardern, Jennifer L. Kuk

**Affiliations:** 1 School of Kinesiology, York University, Toronto, Ontario, Canada; 2 Ontario Ministry of Tourism, Culture & Sport, Toronto, Ontario, Canada; University of Bath, United Kingdom

## Abstract

**Background:**

It is unclear whether the common physical activity (PA) intensity descriptors used in PA guidelines worldwide align with the associated percent heart rate maximum method used for prescribing relative PA intensities consistently between sexes, ethnicities, age categories and across body mass index (BMI) classifications.

**Objectives:**

The objectives of this study were to determine whether individuals properly select light, moderate and vigorous intensity PA using the intensity descriptions in PA guidelines and determine if there are differences in estimation across sex, ethnicity, age and BMI classifications.

**Methods:**

129 adults were instructed to walk/jog at a “light,” “moderate” and “vigorous effort” in a randomized order. The PA intensities were categorized as being below, at or above the following %HRmax ranges of: 50–63% for light, 64–76% for moderate and 77–93% for vigorous effort.

**Results:**

On average, people correctly estimated light effort as 51.5±8.3%HRmax but underestimated moderate effort as 58.7±10.7%HRmax and vigorous effort as 69.9±11.9%HRmax. Participants walked at a light intensity (57.4±10.5%HRmax) when asked to walk at a pace that provided health benefits, wherein 52% of participants walked at a light effort pace, 19% walked at a moderate effort and 5% walked at a vigorous effort pace. These results did not differ by sex, ethnicity or BMI class. However, younger adults underestimated moderate and vigorous intensity more so than middle-aged adults (P<0.05).

**Conclusion:**

When the common PA guideline descriptors were aligned with the associated %HRmax ranges, the majority of participants underestimated the intensity of PA that is needed to obtain health benefits. Thus**,** new subjective descriptions for moderate and vigorous intensity may be warranted to aid individuals in correctly interpreting PA intensities.

## Introduction

It is well established that habitual physical activity (PA) participation is associated with many health benefits [1]–[3]. Some of those health benefits include reduced morbidity and mortality risk, increased energy, stress reduction as well as weight maintenance [4]. However, despite the health benefits associated with PA participation, only one half of Canada’s population is currently active [5], [6]. Physical inactivity is responsible for 9% of global all-cause mortality [7] and it is the fourth leading cause of death [8].

The Canadian government (Health Canada) first published the *Canada’s Physical Activity Guide (CPAG)* in 1998. These original guidelines were updated in 2011 and the basic messages and PA intensity descriptors within the guidelines remained the same. The guidelines recommend the minimum and optimal amounts of PA that Canadians must engage in to accumulate health benefits. The guidelines refer to the well-known inverse relationship between intensity of PA and time [9] specifying that an individual can meet the minimum PA recommendations by engaging in shorter duration PA at vigorous or high intensity or longer duration PA at light intensity [10], [11].

Globally, PA guidelines [12]–[16] use similar intensity descriptors to describe the relative PA intensity as defined by a percentage of age-predicted maximum heart rate (%HRmax). The descriptors used to describe PA intensity, “sedentary,” “light,” “moderate” and “vigorous” are not just used in PA guidelines but they are also used to classify relative exercise intensities using %HRmax. It is unclear if these common descriptors align with the %HRmax ranges used in the literature consistently between sexes, ethnicities, age categories and across body mass index (BMI; kg/m^2^) classifications. This information may be critical for public health officials in order to properly frame messages regarding PA participation for all populations. Therefore, the objectives of this study are to determine whether individuals understand the commonly used PA intensity descriptors in PA guidelines and properly self-select light, moderate and vigorous intensity PA. The second objective is to determine if there are differences in the understanding of PA intensity descriptors by age, sex, ethnicity or BMI class.

## Materials and Methods

### Ethics Statement

All participants provided written informed consent prior to participation in the study and all procedures were approved and conducted in accordance with the ethical guidelines of the York University Institutional Review Board.

### Participants

Participants aged 18–64 were recruited from York University between September 2009 and December 2012. Participants were excluded from the analysis if they had missing data for age, ethnicity, BMI, light, moderate or vigorous intensity measured heart rates (n = 11). This left a total sample of 129 participants consisting of (n = 39) men and (n = 90) women.

### Demographics and Questionnaires

Participants completed a variety of lifestyle, demographic and medical history questionnaires, and were screened using the Physical Activity Readiness Questionnaire (PAR-Q). Following the completion of the VO_2_ peak protocol the study participants completed a questionnaire regarding the ease of understanding and use of the CPAG employing a Likert measurement scale from 1 (easy) to 5 (hard). In addition, participants were asked if they would use the PA guidelines and if they meet the minimum PA guidelines.

### Self-Estimate of PA Intensity

Participants were instructed to walk and/or jog on the treadmill at a speed which they felt corresponded to the “light,” “moderate” and “vigorous ” PA intensity descriptor, in a randomized order. The investigator read the following descriptor definitions to the participants. For light effort: “you are starting to feel warm and you have a slight increase in breathing rate”; for moderate effort: “you are warmer and you have a greater increase in breathing rate”, and; for vigorous effort: “you are quite warm and more out of breath”. While wearing a Polar heart rate (HR) monitor, participants went at each self-selected speed for 2 to 3 minutes to allow for HR to reach steady state. HR (bpm) and speed (mph) were recorded for each of the intensities. Participants were then asked to walk at the minimal pace that they believed would provide health benefits for 3 minutes. The HR, speed and distance (miles) were recorded by the investigator.

### Peak VO_2_ Peak Exercise Test

VO_2_peak was assessed using a modified Balke treadmill protocol. Prior to February 2012, VO_2_peak was calculated using the formula 4.702–[0.0924*weight (kg)]+[6.191*speed (mph)]+[1.311*gradient]+[2.674 for males only] [17] (N = 94), and directly measured using a Cardio Coach CO2 9000 metabolic cart (KORR, Salt Lake City, UT, USA) after its acquisition in February 2012 (N = 35). Participants were asked to choose a speed that corresponded to a 6 on a rating of perceived exertion scale (RPE), where 0 was at rest and 10 was maximal exertion [18]. Given the wide age range of participants, it was more appropriate to use a RPE scale of 0–10 as opposed to the commonly used 6–20 Borg scale. The speed remained constant for the entire test and the incline was adjusted by 2% after the first minute and 1% for every minute thereafter until the participant reached volitional exhaustion or if there were safety concerns. Participants were given verbal encouragement consistently by the testers. The VO_2_peak tests were performed 5–10 minutes following the self-estimate of intensity protocols on the treadmill, allowing the participant to bring their heart rate down to resting state.

A VO_2_peak was considered to be acceptable when: A) presence of a plateau in VO_2_ was observed, B) RER >1.0, or C) HR was greater than 90% of the study participant’s age-predicted maximum HR. For participants with acceptable VO_2_ peak tests, the HR during light, moderate and vigorous intensity PA bouts were expressed both as a percent of the participants’ HR peak achieved during the VO_2_ peak treadmill test (% HRpeak) in addition to their age-predicted maximum HR (220-age) (%HRmax). For participants without a measured or an acceptable VO_2_peak test, only %HRmax was used.

The measured steady state HR for each self-selected exercise bout was categorized as being below, at, or above the exercise intensity cut-off ranges of 50–63%HRmax (or %HRpeak) for light effort, 64–76%HRmax (or %HRpeak) for moderate effort and 77–93%HRmax (or %HRpeak) for vigorous effort [19], [20].

### Statistical Analyses

Continuous variables are expressed as means (± SD). Chi-square tests were used to compare the number of individuals who identified exercise intensities below, at, or above the defined %HRmax (or %HR peak) intensities in the whole sample and by sex, ethnicity (White versus Non-White: African, South Asian, Chinese, Latin American, Arab, other), age (young: <30 years old versus middle-aged/old ≥30 years old) and BMI (kg/m^2^; underweight/normal weight: ≤18.5–24.9 versus overweight/obese: ≥25) classifications. Paired t-tests were used to determine the differences between %HRpeak and %HRmax values in the subset who had acceptable VO_2_ peak tests. All statistical analyses were performed using SAS v9.3. Statistical significance was set at alpha <0.05.

## Results

Participant characteristics are presented in **Table 1** **.** The majority of the study participants were young, normal weight women with poor cardiovascular fitness. Eighty percent of participants indicated that the CPAG was easy to understand. Furthermore, sixty-five percent of the participants said they would use the CPAG and 57% of participants reported that they met the minimum PA guidelines of 150 minutes of moderate intensity PA per week.

**Table 1:** Participant Characteristics.

Variable(n)	Men(39)	Women(90)	P Value
Age (y)	20.8±5.3	24.8±10.9	P = 0.0313
White (%)	43.6	55.6	P = 0.2116
BMI (kg/m^2^)	24.0±3.4	23.6±6.1	P = 0.9304
Underweight/Normal Weight (%)	69.2	70.0	
Overweight/Obese (%)	30.8	30.0	
VO_2_ Max (mL O_2_/kg/min)	40.6±8.5	34.0±7.6	P<0.0001
Desire to use CPAG (%)	63.2	65.6	P = 0.7952
Self-reported meeting PA recommendations (%)	71.4	50.0	P = 0.0336

Data are presented as means ± SD, by prevalence (%) or range (minimum-maximum).

BMI: Body Mass Index.

CPAG: Canada’s Physical Activity Guide.

PA: Physical Activity.

The HR responses during the self-selected light, moderate and vigorous effort walking treadmill speeds corresponded to the following %HRmax ranges: 51.5±8.3, 58.7±10.7 and 69.9±11.9, respectively. When directed to walk at a pace that the participants thought would provide health benefits, they walked at an intensity of 57.4±10.5%HRmax; less than the moderate effort range (64–76%HRmax). Specifically, 52% percent walked at a light effort pace (56.8±3.8%HRmax), 19% walked at a moderate effort pace (67±5.5%HRmax) and only 5% walked at a vigorous effort pace (85.3±9.6%HRmax). The %HRmax that the young and middle-aged/old categories thought would provide health benefits differed (P<0.05). Younger individuals walked at an intensity of 56.6±9.4%HRmax (range: 38.4–84.7%HRmax) whereas middle-aged and older individuals walked at an intensity of 59.5±13.5%HRmax (range: 35.4–103.5%HRmax).

**Figure 1:**
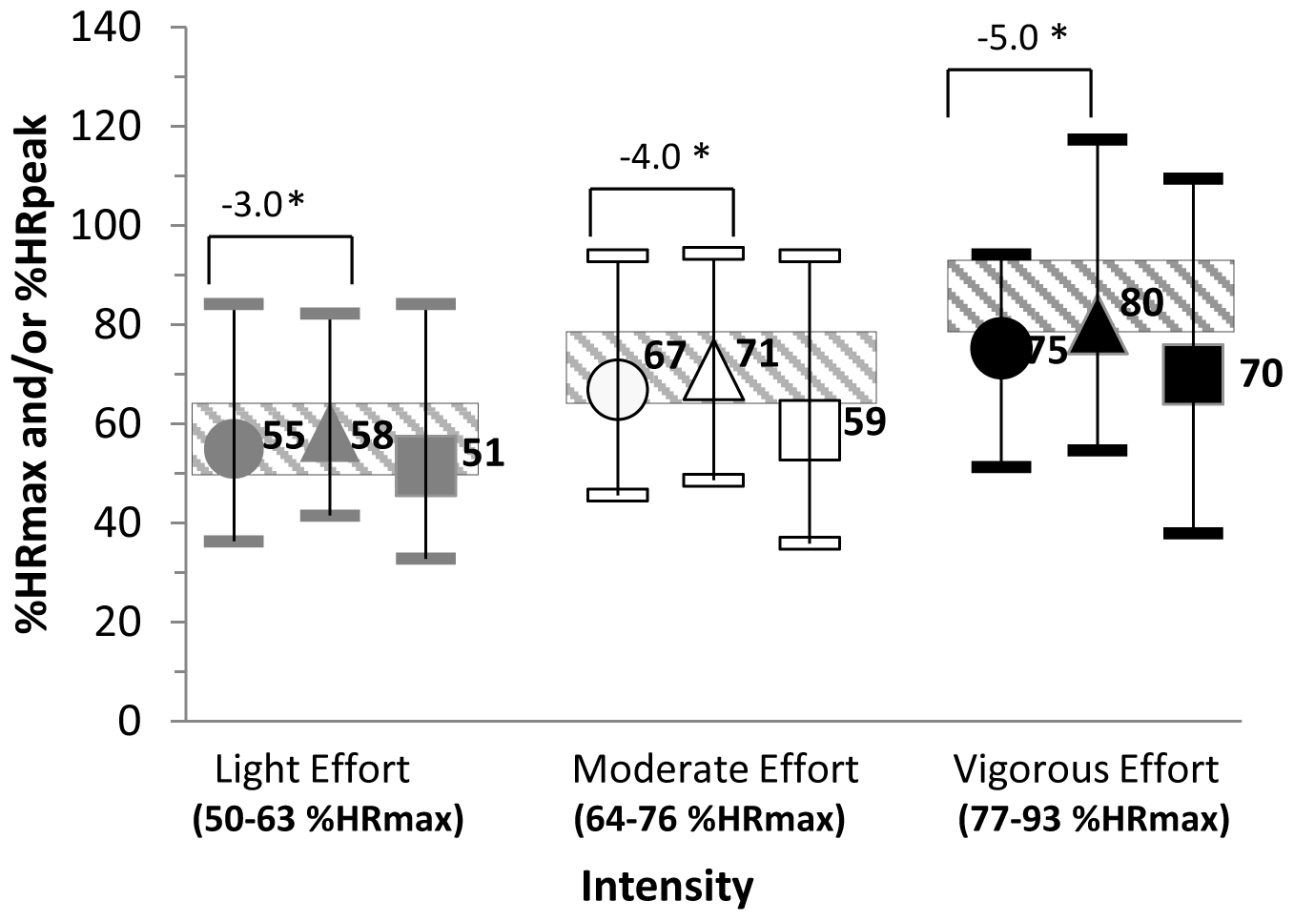
Measured %HRmax (n = 129) and %HRpeak (n = 35) during Light, Moderate and Vigorous Effort Treadmill exercise. * Significant difference at P<0.0001. •%HRmax = Observed HR/(220-age) (sub-sample, n = 35). ▴%HRpeak = Observed HR/(HRpeak VO_2_ treadmill test) (sub-sample, n = 35). ▪%HRmax = Observed HR/(220-age) (population, n = 129). Cross-hatched boxes = %HRmax range: 50–63% light effort; 64–76% moderate effort; 77–93% vigorous effort.

The age-predicted maximum HR formula (220-age) yielded significantly higher HRmax values than the observed HR peak from the incremental to maximal treadmill test (P<0.0001; 190 bpm versus 178 bpm, respectively). This translated into a higher %HRpeak compared to %HRmax values during light, moderate and vigorous effort PA **(** **Figure 1** **)** which resulted in mean differences (%HRmax-%HRpeak) of −3.4% for light effort, −4.0% for moderate effort and −4.9% for vigorous effort (n = 35).

**Figure 2:**
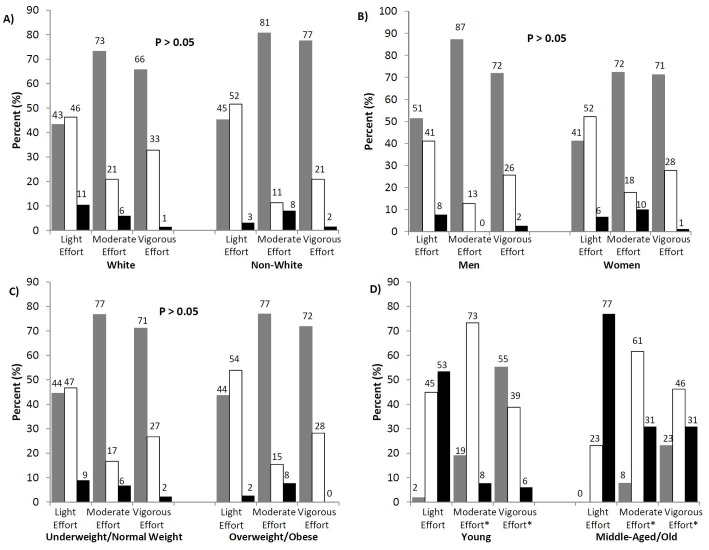
Accuracy of the self-estimated PA Intensity across A) Ethnicity, B) Sex, C) Body Mass Index (BMI) and Age (D) as confirmed by measured %HRmax. *Significant difference at P<0.05. Grey bars- below %HRmax range. White bars- within %HRmax range. Black bars- above %HRmax range. %HRmax range: 50–63% light effort; 64–76% moderate effort; 77–93% vigorous effort.

There was no difference by ethnicity **(** **Figure 2A** **)**, sex **(** **Figure 2B** **)** or BMI categories **(** **Figure 2C** **)** in the understanding of light, moderate and vigorous intensity (P>0.05). Most participants correctly estimated light effort PA and underestimated moderate and vigorous effort PA. There was no difference in the understanding of light effort PA (P>0.05) between the age groups, with both young and middle-aged participants overestimating light effort PA. However, there was a difference in the understanding of moderate and vigorous effort between the young and middle-aged/old **(** **Figure 2D** **)**. Although the majority of participants in both age groups correctly estimated moderate effort, younger individuals estimated moderate effort to be a lower %HRmax compared to older individuals (P<0.05). Similarly, younger individuals underestimated vigorous intensity whereas middle-aged individuals correctly estimated vigorous effort (P<0.05).

## Discussion

The findings from this study suggest that the majority of young and middle-aged to old adults underestimate the intensity of PA that is required to attain health benefits. Participants accurately estimated light effort PA and underestimated moderate and vigorous effort PA, and there appear to be no differences by sex, ethnicity or across BMI classifications in the understanding of the commonly used PA intensity descriptors. However, middle-aged individuals appear to have a better understanding of moderate and vigorous intensity than younger individuals. Therefore, the current subjective PA intensity descriptions may need to be enhanced in order to align with the associated relative PA intensities using %HRmax.

Previous research suggests that even inexperienced adults correctly identify moderate intensity walking when information related to speed of walking and heart rate is linked to how their body feels when exercising [21]. Spelman et al (1993) [22] also suggest that the self-selected exercise intensity of habitual exercise walkers approximates moderate intensity and meets the minimum American College of Sports Medicine recommendation for improving aerobic fitness. The findings from this current study suggest that the current descriptors commonly used to describe PA intensity in many PA guidelines, lead to the underestimation of moderate and vigorous effort PA, and underestimate the PA intensity required to achieve minimum health benefits. These differences may reflect secular changes in the understanding of the PA intensity terms or simply differences in study cohorts. Thus, future studies are needed to verify these findings. This is extremely important given the efforts to harmonize different expressions of relative intensities for aerobic physical activity participation [19].

The descriptors that describe PA intensity are not just used in PA guidelines but they are also used to classify relative exercise intensities using %HRmax. However, much of the research literature suggests that using individuals’ peak HRs from maximal exercise tests to prescribe exercise is more accurate than using the age-predicted maximum formula [23]–[25] as it takes into account individual differences in maximal HR [24], [26]. However, incremental to maximum exercise testing is expensive and not readily accessible. Thus, the majority of individuals will need to rely on age predicted maximum formula of 220-age to calculate the proper PA intensity. This current study demonstrates that though statistically significant, the differences when using %HRpeak versus %HRmax were ≤5%. This magnitude of difference has minimal clinical relevance, and thus, we suggest that %HRmax can adequately be used to identify PA intensities.

A wealth of evidence clearly demonstrates the benefits of participating in PA [1], [4], [27], [28], however despite this research, the majority of Canadians are still physically inactive [5], [6]. In a recent publication with accelerometer data from Canada’s Health Measures Survey, only 15% of Canadians are participating in 150 minutes of moderate to vigorous PA per week in 10 minute bouts and only 53% of Canadians participate in 30 minutes of moderate to vigorous PA at least one day per week [6], meaning that the majority of Canadians are not active enough for minimum health benefits. However, if individuals are overestimating the intensity of PA that they are engaging in, the problem of physical activity may in fact be even larger.

Health Canada first published the CPAG in 1998 as a national initiative to help Canadians become physically active and incorporate PA as a part of their lifestyle [20]. To align with the changes in the global PA guidelines, updated evidence-informed PA guidelines were released in early 2011 by the Canadian Society for Exercise Physiology and the Public Health Agency of Canada. These guidelines revised the recommendation for achieving minimum health benefits in Canadians aged 18–64 years from 30 to 60 minutes of moderate effort PA or 20 to 30 minutes of vigorous effort PA on all or most days of the week to 150 minutes of moderate-to-vigorous aerobic PA per week, in bouts of 10 minutes or more [29]. To describe intensity of PA, the revised guidelines use the same descriptors and corresponding objective physiological values of intensity defined by specific ranges of %HRmax. The commonly used PA intensity descriptors in the guidelines appear to be sufficient for explaining light intensity, but do not appear optimal for relaying the correct intended physiological intensity for moderate and vigorous PA intensities. It is unclear currently how these differences in understanding might influence physical activity participation, but may lead individuals to mistakenly interpret that they are sufficiently active to achieve health benefits.

Several limitations must be taken into account. Despite having a large age range, a large proportion of participants in this study were younger with the median age being 20 years old. Therefore, the results should be verified in older populations. Further, we are unaware if unknown differences in the populations recruited may have altered the observations seen here. Secondly, we asked participants to walk at a speed they thought would provide health benefits, however we are unable to know whether these individuals can exercise at this pace for sufficiently long periods of time to obtain the desired health benefits. Third, it is unknown if our participants would use the PA intensity descriptors in the CPAG similarly when using other types of exercise equipment such as a bike or elliptical or even non-treadmill walking. Lastly, this study was initiated prior to the release of the revised 2011 CPAG, and although the same PA intensity descriptors were used, the framing and context of the revised guidelines may alter how these descriptors are interpreted. In addition, it is unclear as to whether the underestimation of moderate and vigorous intensity PA are as a result of the poor cardiovascular fitness and may be lack of exercise experience observed in our sample. Future studies should assess intensity estimation in samples with greater fitness to confirm our findings.

In conclusion, this is the first study to determine that adults of different sexes, ethnicities and BMI classifications underestimate moderate and vigorous intensity PA, and underestimate the PA intensity recommended for health. Given the difficulties in understanding moderate and vigorous effort PA, new subjective descriptions for moderate and vigorous intensity may be warranted to aid individuals with the understanding of PA intensity.
